# Association Between Chronic Obstructive Pulmonary Disease and Low Muscle Mass in Korean Adults

**DOI:** 10.3390/jcm14041134

**Published:** 2025-02-10

**Authors:** Do-Youn Lee

**Affiliations:** College of General Education, Kookmin University, Seoul 02707, Republic of Korea; triptoyoun@kookmin.ac.kr; Tel.: +82-02-910-5540

**Keywords:** COPD, RPD, pulmonary disease, low muscle mass, sarcopenia

## Abstract

**Background/Objectives**: Chronic obstructive pulmonary disease (COPD) and restrictive pulmonary disease (RPD) are prevalent in older adults and are associated with systemic inflammation, oxidative stress, and reduced physical activity, all of which may contribute to low muscle mass (LMS)—a loss of skeletal muscle mass and function. This study investigates the association between COPD, RPD, and sarcopenia using data from the Korea National Health and Nutrition Examination Survey (KNHANES). **Methods**: A cross-sectional analysis was conducted with 8980 participants aged 40 years and older from the 2008–2011 KNHANES. LMS was defined using the Asian Working Group for Sarcopenia (AWGS) criteria, based on appendicular skeletal muscle mass adjusted for height squared (ASM/height^2^). Pulmonary diseases were identified via spirometry, and logistic regression models were used to assess associations, adjusting for demographic, lifestyle, and clinical factors. **Results**: The prevalence of LMS was highest among participants with COPD (37.3%), compared to those with RPD (25.3%) and normal pulmonary function (25.9%). COPD was significantly associated with LMS after adjusting for confounders (OR: 1.543, 95% CI: 1.246–1.910). However, no significant association was observed between RPD and LMS (OR: 1.225, 95% CI: 0.997–1.505). **Conclusions**: This study demonstrated that LMS was independently associated with COPD but not with RPD, even after adjusting for confounding variables.

## 1. Introduction

Chronic obstructive pulmonary disease (COPD) and restrictive pulmonary disease (RPD) are critical public health concerns that substantially influence the geriatric demographic [1,2]. These pathologies result in respiratory impairment, diminished physical capabilities, and a lowered quality of life [3,4]. Sarcopenia, often used interchangeably with low muscle mass (LMS), refers to the loss of muscular mass, efficiency, and skeletal muscle function, which has been associated with a number of adverse effects such as mortality, increased chances of hospitalization, and frailty, and is even postulated to be related with lung disease [4,5]. In Korea, particularly in the elderly segment of the population, which has been noted to suffer the most from sarcopenia, the incidence of sarcopenia is increasing rather rapidly; thus, it has become a serious public health concern [6].

COPD is a progressive disease defined by irreversibly impaired airflow, usually caused by inhalation of environmental toxins or chronic tobacco-based inflammation [7]. RPD is defined as decreased lung expansion due to interstitial lung disease, obesity, or neuromuscular disorders [8]. LMS can be caused by aging, chronic inflammation, sedentary lifestyle, etc. [9]. Both pulmonary disease and LMS are known to be aggravated by metabolic disturbance or low-grade inflammation and inactivity, which lead them to be more morbid [6,10].

Pulmonary diseases and sarcopenia have many common etiological factors, such as chronic systemic inflammation, oxidative stress, physical inactivity, and nutritional deficiency [7,10,11,12,13]. Sarcopenia muscular atrophy may also contribute to respiratory muscle weakness, reducing pulmonary function [12,14]. On the other hand, impaired pulmonary function can limit physical activity, which can expedite muscle loss [7,15]. This bi-directional association demonstrates the importance of understanding their interaction to enhance the clinical outcomes in affected individuals [7,12].

Until now, although more and more researchers have focused on the relationship between pulmonary diseases and LMS, most studies did not take fundamental confounding factors like comorbidities and lifestyle behaviors into account in their analyses [16]. Furthermore, a large number of research studies were limited to specific cohorts or did not differentiate between COPD and RPD [16,17]. There is still a need for large-scale studies using nationally representative data to describe these relationships in more detail.

To address this, the current study aimed to determine the correlation between LMS and pulmonary disease (COPD and RPD) based on data from the Korea National Health and Nutrition Examination Survey (KNHANES). Using a nationally representative sample, the study seeks to show that the association is independent of demographic, behavioral, and clinical factors. The purpose of the study was to assess the risk of LMS in patients with respiratory diseases to take this into account when planning preventative measures for this condition.

## 2. Materials and Methods

### 2.1. Study Design and Participants

**Figure 1:**
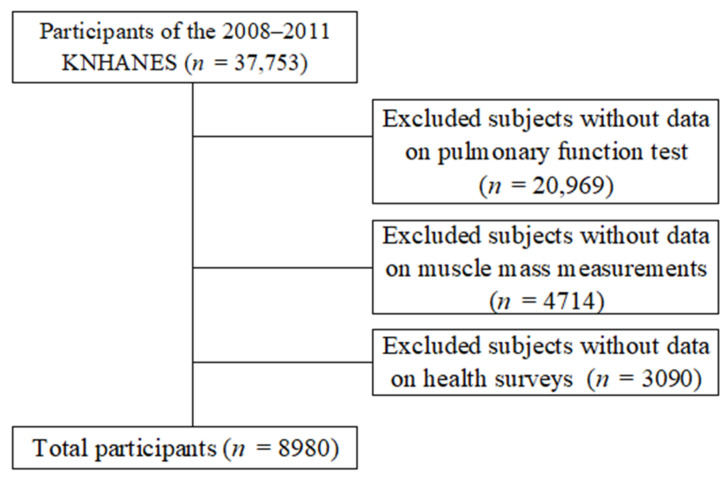
Selection of participants from Korea National Health and Nutrition Examination Survey 2008–2011.

This is a cross-sectional study based on the data from the 2008–2011 Korea National Health and Nutrition Examination Survey. Among the total participants, 37,753 were excluded if they did not participate in the pulmonary function test, were under 40 years old, lacked LMS measurement data (4714 individuals), or did not complete the health survey (3090 individuals). This left 8980 participants for analysis (Figure 1).

### 2.2. Measurements of Variables

#### 2.2.1. Covariates

##### Socio-Demographic Characteristics

Demographic variables included sex, age, education level, marital status, and personal income level. Age was grouped into 40–49, 50–59, 60–69, and 70 years or older. Education level was categorized as low or high depending on whether participants graduated from high school. Marital status was defined based on current spousal cohabitation. Income levels were divided into quartiles according to average monthly personal income.

##### Health-Related Characteristics

BMI was calculated as weight in kilograms divided by height in meters squared (kg/m^2^) and classified into underweight, normal, overweight, and obesity categories. Blood pressure was measured using a mercury gauge after a 5 min stabilization period, with three readings taken at 30 s intervals. Hypertension was defined as systolic blood pressure ≥ 130 mmHg, diastolic blood pressure ≥ 85 mmHg, or current use of antihypertensive medication. Blood samples were collected after at least 8 h of fasting and analyzed within 24 h using a Hitachi Automatic Analyzer 7600 (Tokyo, Japan). Hyperglycemia was defined as fasting blood glucose ≥ 100 mg/dL or current diabetes medication use. Hypertriglyceridemia was characterized by triglyceride levels ≥ 150 mg/dL. Low HDL-C was defined as <40 mg/dL for men and <50 mg/dL for women. Abdominal obesity was determined by a waist circumference ≥ 90 cm for men and ≥85 cm for women [18].

##### Lifestyle Characteristics

Smoking status was categorized as current daily smoking, occasional smoking, past smoking (formerly smoked but not currently), or non-smoking. Drinking status was divided into current drinking (at least once a month) and non-drinking. Physical activity included both aerobic and muscular exercises. Aerobic exercise was defined as moderate-intensity activity for at least 2 h and 30 min per week, high-intensity activity for at least 1 h and 15 min per week, or a mix of the two. Muscular exercise was defined as strength training performed at least twice per week [19].

#### 2.2.2. Measurement of Pulmonary Function

A spirometer (model 2130; SensorMedics, Yorba Linda, CA, USA) was used to measure pulmonary function. FVCp values, which are the predicted values of FVC, respectively, were used as continuous variables [20]. Based on their spirometry patterns, the participants were divided into three groups: normal (FEV1/FVC ≥ 0.70, FVC ≥ 80% predicted), restrictive (FVC < 80% predicted, FEV1/FVC ≥ 0.70), and obstructive (FEV1/FVC < 0.70).

#### 2.2.3. Measurements of Skeletal Muscle Mass

To ascertain muscle mass and body composition, certified technicians employed DXA (Discovery QDR 4500 W, Hologic Inc., Belford, MA, USA). Prior to the test, participants were put in a supine position and fasted. It was understood that skeletal muscles were not bone or fat tissues. The total of the skeletal muscle masses in the arms and legs, as determined by DXA, was known as appendicular skeletal muscle mass, or ASM. ASM (kg) divided by height in meters squared (m^2^) yielded the skeletal muscle mass index (SMI). As advised by the Asian Working Group for Sarcopenia, LMS was defined as SMI < 7.0 kg/m^2^ for men and <5.4 kg/m^2^ for women [21].

### 2.3. Data Analysis

The data analysis was conducted using SPSS 28.0 for Windows (IBM, Armonk, NY, USA). To account for the multistage, complex probability sampling design, survey weights were applied following standard recommendations. Given the stratified, random, and cluster sampling approach, appropriate weighting adjustments were made. Data are presented as absolute counts and estimated percentages with standard errors (SEs). Group differences in demographic and clinical characteristics based on pulmonary disease and LMS were assessed using the χ^2^ test or Student’s *t*-test. Multivariate logistic regression was employed to examine the relationship between skeletal muscle mass and pulmonary disease, with odds ratios (ORs) and 95% confidence intervals (CIs) calculated accordingly. A *p*-value of <0.05 was considered statistically significant.

## 3. Results

### 3.1. Characteristics of Participants According to Pulmonary Diseases

The characteristics of COPD, RPD, and normal group participants are presented in Table 1. The LMS prevalence was significantly highest for participants with COPD (37.3%), followed by those with RPD (25.3%), and then the normal group (25.9%). The COPD group had an older participant base, predominantly male, while normal group participants were the youngest on average and included the most university-educated individuals. The COPD group also showed the highest rates of active smoking, low BMI, and elementary-level educational attainment, whereas higher obesity rates and greater incomes were more representative of the RPD group. Regarding lung function parameters, as expected, these were lowest in the COPD group. Comorbid hypertension, diabetes, and abdominal obesity were more significant in COPD and RPD subjects than in those in the normal group. Lifestyle factors varied more significantly in resistance exercise participation; the COPD group reported the highest proportion of individuals who never engaged in resistance exercises, though aerobic exercise participation did not show significant differences between the groups.

### 3.2. Characteristics of Participants According to LMS

Table 2 shows the characteristics of the study participants according to sarcopenia status. The history of COPD and RPD was higher in the LMS group (15.3% and 10.2%, respectively) compared to the normal group (9.6% and 11.2%). Participants with LMS were older and included a lower proportion of men compared to those in the normal group. In terms of education, the LMS group had a higher percentage of individuals with lower educational attainment (elementary and middle school levels) and a lower percentage of university graduates. There was a difference in marital status, and the proportion of participants in the LMS group who did not live with their spouse was higher. BMI distribution indicated that individuals with LMS were more likely to be in the normal weight category, while overweight and obesity rates were higher in the normal group. Regarding lifestyle factors, participants in the LMS group reported lower smoking and drinking rates and less frequent participation in aerobic and resistance exercises. Lung function measures, including FVC, FEV1, and FEV1/FVC, as well as the skeletal muscle index (SMI), were notably lower in individuals with LMS. Lastly, the prevalence rates of comorbidities such as hypertension, diabetes, high triglycerides, low HDL-C, and abdominal obesity were significantly lower in the LMS group compared to the normal group.

### 3.3. Odds Ratios for COPD and RPD According to LMS Status

Table 3 presents the association between pulmonary diseases and LMS. The crude model showed a significant association between COPD and LMS, whereas RPD had no significant association. In Model 1, adjusted for demographic factors, COPD displayed a positive association with LMS (OR: 1.791, 95% CI: 1.472–2.178), while RPD remained non-significant. In Model 2, after adjusting for BMI, lifestyle factors, and exercise, the association of COPD with LMS weakened (OR: 1.519, 95% CI: 1.225–1.883), with no significant relationship observed for RPD. In the fully adjusted Model 3, which included comorbidities, COPD maintained a significant association with LMS (OR: 1.543, 95% CI: 1.246–1.910). For RPD, there was still no significant association.

### 3.4. Odds Ratios for COPD and RPD According to SMI

Table 4 presents the association between pulmonary diseases and the skeletal muscle index (SMI). In the crude model, both COPD and RPD showed a significant association with the SMI. In Model 1, adjusted for demographic factors, COPD was inversely associated with the SMI (OR: 0.552, 95% CI: 0.494–0.617, *p* < 0.001), while RPD’s association with it was not significant (OR: 1.053, 95% CI: 0.947–1.172, *p* = 0.338). After further adjustment for BMI, lifestyle factors, and physical activity in Model 2, the association between COPD and the SMI remained significant (OR: 0.723, 95% CI: 0.628–0.832, *p* < 0.001), whereas RPD’s association remained non-significant. In Model 3, which included comorbidities, COPD maintained a strong inverse association with the SMI (OR: 0.713, 95% CI: 0.619–0.821, *p* < 0.001), while RPD showed no significant association (OR: 0.909, 95% CI: 0.793–1.042, *p* = 0.170). These results indicate that higher muscle mass is associated with a lower risk of COPD, while no significant relationship was found for RPD.

### 3.5. Dose–Response Relationship Between Pulmonary Function and SMI

**Figure 2:**
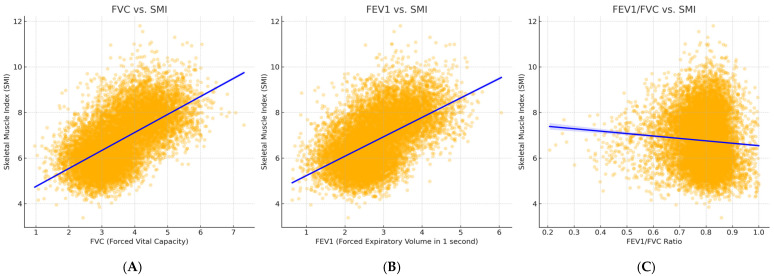
Association between pulmonary function parameters and the skeletal muscle index (SMI). Scatter plots with fitted regression lines show the relationship between (**A**) forced vital capacity (FVC), (**B**) forced expiratory volume in 1 s (FEV1), and (**C**) FEV1/FVC ratio and the SMI. The blue regression lines indicate a positive correlation between pulmonary function and SMI.

To explore the dose–response relationship between pulmonary function and skeletal muscle mass, we performed a weighted regression analysis (Figure 2). As shown in Figure 2A, FVC is positively correlated with the SMI, indicating that lower lung volume is associated with greater muscle loss. Figure 2B shows a similar trend for FEV1, suggesting that airflow limitation, particularly in COPD, may contribute to muscle wasting. Figure 2C further demonstrates that a lower FEV1/FVC ratio is linked to a lower SMI, reinforcing the connection between COPD and LMS.

## 4. Discussion

The main results of this study were that COPD was significantly associated with LMS in Korean adults after adjusting for numerous covariates. The association was particularly strong in COPD and not in RPD. In our study, the prevalence of LMS was significantly higher in participants with COPD (37.3%) than in participants with RPD (25.3%) and the normal group (25.9%). In addition, COPD was found to be robustly correlated with LMS when controlling for confounders; the OR was 1.543 (95% CI: 1.246–1.910) in the fully adjusted model. Conversely, RPD did not exhibit a significant association with LMS (OR 1.225, 95% CI: 0.997–1.505).

Furthermore, this study reported a dose–response association of pulmonary function with the SMI (Figure 2), supporting a potential biological association between respiratory impairment and sarcopenia. These findings showed significantly lower FVC, FEV1, and FEV1/FVC with reduced muscle mass and suggest that even mild declines in lung function may be linked to LMS. These observations aligned with previous studies showing that impaired respiratory function is closely linked to muscle wasting, particularly in COPD patients [22]. The observed dose–response relationship emphasizes the potential role of early pulmonary dysfunction in accelerating muscle loss, thereby marking the key target of timely intervention with pulmonary rehabilitation.

The observed correlation between LMS and COPD may be explained by a number of mechanisms. First, a hallmark of COPD, systemic inflammation, stimulates muscle catabolism by activating pro-inflammatory cytokines such as interleukin-6, interleukin-1β, interleukin-8, and tumor necrosis factor-α. These cytokines then trigger NF-κB signaling pathways, which results in decreased protein synthesis and protein degradation in skeletal muscle [22,23]. Increased numbers of activated neutrophils and macrophages worsen this inflammatory response by releasing proteolytic enzymes that further degrade muscle [24,25]. Second, skeletal muscle damage becomes more severe due to oxidative stress caused by long-term respiratory dysfunction, which is described by an imbalance between oxidant production and antioxidant defenses [26,27]. Reactive oxygen species, primarily from mitochondria and nicotinamide adenine dinucleotide phosphate oxidase, are elevated in COPD patients, leading to oxidative damage to skeletal muscle proteins, lipids, and DNA [28]. This impairment leads to muscle atrophy and dysfunction, which is made worse by decreased antioxidant capacity, which includes lower levels of glutathione and superoxide dismutase [27,29]. Third, hypoxia associated with COPD might impact mitochondrial function, which could decrease muscle endurance and accelerate muscle wasting [30]. This transition leads to a reduction in muscle endurance and an increased susceptibility to fatigue when coupled with other mitochondria dysfunction [31]. The hypoxia-inducible factor (HIF)-1α activation that takes place under a hypoxic local environment also promotes a fiber type switch in muscle from an oxidative to a glycolytic profile, which adversely affects muscle function [32]. Fourth, daily ambulation in COPD patients decreases by about 400–500 steps, which leads to muscular incapacity, and muscle deconditioning due to physical inactivity in COPD patients leads to further reduced levels of activity [33,34]. This muscular degradation fosters a negative feedback loop that speeds up muscular degradation by triggering proteolytic pathways, like ubiquitin–proteasome systems and autophagy–lysosome routes [33,35,36,37]. Fifth, a major feature of COPD is hormonal dysregulation, with elevated levels of catabolic hormones such as cortisol, and decreased levels of anabolic hormones such as growth hormone, testosterone, and IGF-1 [22,38]. An imbalance in hormones combined with reduced responsiveness of skeletal muscle to anabolic stimuli (anabolic resistance) impedes the processes involved in muscle maintenance [38,39].

The association of LMS with COPD has been seriously investigated in previous studies [13,16,40,41]. One other study recognized LMS as an independent predictor of COPD, with an OR of 5.982 (95% confidence interval, 1.576–22.704) [40]. Such results closely match those we obtained, suggesting that such an association is consistent across different populations. Another systematic review and meta-analysis study found that the prevalence of LMS among patients with COPD was 55% [11]. Indeed, similar patterns have been shown by studies conducted among Asian populations, thereby affirming the generalizability of our findings in the Korean context [42,43]. The comparable effect sizes in different ethnic populations suggest that LMS’s association with COPD is a universal phenomenon and a reflection of fundamental biology, not attributable to a specific population.

One interesting thing found in this study is that, unlike COPD, RPD was not significantly associated with LMS. This discrepancy suggests that the role of obesity in the limitation of lung function may be a partial explanatory factor. As can be seen from Table 3, the inclusion of BMI in Model 2 significantly increases the OR value of RPD. This may mean that obesity contributes to the limitation of lung capacity regardless of the underlying pathology of the lungs and that BMI is a mediating factor in the association between RPD and LMS [44]. Considering that obesity can be a risk factor for RPD and a potential confounding factor for LMS, future studies should consider a more detailed body composition assessment to explain the interactions between lung function and obesity and muscle mass loss.

Although our results explain an apparent association between COPD and LMS, several studies have reported inconsistent results. A previous study found no major association between mild COPD and LMS, suggesting that the disease’s severity could have a moderating effect on this association [41]. These inconsistencies may be ascribed to various factors, encompassing methodological disparities in LMS evaluation, differing classifications of COPD severity, and population-specific attributes. A significant contradiction is evident in a preceding study that indicated a correlation between LMS and RPD rather than COPD [17]. Such discrepancies are probably due to differences in diagnostic criteria for LMS. Our study was based on the Asian Working Group for Sarcopenia (AWGS) definition of LMS, with appendicular skeletal muscle mass (ASM) adjusted for height squared (ASM/height^2^), while the previous study followed the definition of the European Working Group on Sarcopenia in Older People 2 (EWGSOP2), where ASM is adjusted for body mass index (ASM/BMI). These observed methodological differences highlight the need for the standardization of these parameters when assessing LMS at the time of study design.

The results of this study are pertinent and contribute to the body of literature exploring the relationship between COPD and LMS. We first provide robust epidemiological evidence of the association in the Korean population, utilizing nationwide representative datasets obtained from KNHANES. Second, our detailed investigation of both COPD and RPD provides an unprecedented perspective on the specificity of the relationship between pulmonary diseases and LMS. Finally, our use of a large, nationally representative sample of patients enhances the generalizability of our findings. The robustness of our findings is bolstered by our stringent diagnostic criteria for both COPD and LMS and by extensive adjustment for potential confounders. The inclusion of both COPD and RPD enables the comparison of their relative associations with LMS and, therefore, offers insights into disease-specific relationships.

In addition to the strengths of this study, several limitations have to be considered. First, the cross-sectional design of our data does not allow for assessing causality between COPD and LMS. Second, our evaluation of LMS was limited to muscle mass, and did not include measurements of muscle function or physical performance, both essential elements of contemporary definitions of LMS. Third, while physical activity was measured using self-reported aerobic and resistance exercise data, the lack of accelerometer-based activity measurement reduces the precision of activity quantification. Fourth, dietary intake, a key determinant of muscle mass, was not included as a covariate in the analysis. Given the important role of protein and overall nutrient intake in muscle maintenance [45], this unmeasured factor may contribute to residual confounding. Lastly, some information about severity and duration of COPD was missing, limiting our capacity to assess dose–response relationships. Additionally, the dataset did not specify whether FEV1/FVC values were measured before or after bronchodilator administration, which may introduce some misclassification. While this criterion is commonly used in epidemiological studies, future research incorporating post-bronchodilator spirometry is needed to validate these findings.

## 5. Conclusions

This study aimed to examine the association between LMS and pulmonary diseases (COPD and RPD) in Korean adults using nationally representative data. After adjusting for confounding variables, LMS was independently associated with COPD but not with RPD.

## Figures and Tables

**Table 1:** Characteristics of participants according to pulmonary diseases.

Factors	Categories	COPD (*n* = 1131)	RPD (*n* = 1071)	Normal (*n* = 6778)	*p*
		M or %	M or %	M or %	
Prevalence LMS		37.3	25.3	25.9	<0.001
Age (y)		61.89 ± 0.38 ^a^	57.64 ± 0.45 ^b^	52.38 ± 0.16 ^c^	<0.001
Sex	Male	73.4	55.4	45.1	<0.001
Education	Elementary	22.8	15.5	12.4	<0.001
	Middle	28.3	21.1	19.2	
	High	23.6	22.4	25.5	
	University	25.4	40.9	42.9	
Marital status	With	82.8	82.4	86.5	<0.001
	Without	17.2	17.6	13.5	
Personal income	Q1 (lowest)	27.8	24.1	23.7	0.009
	Q2	28.2	26.5	26.1	
	Q3	22.4	22.5	26.5	
	Q4 (highest)	21.6	26.9	23.7	
BMI	Low	4.2	2.8	1.0	<0.001
	Normal	69.0	47.5	61.3	
	Overweight	25.2	42.2	34.6	
	Obesity	1.6	7.5	3.1	
Smoking status	Current	54.9	40.5	32.8	<0.001
	Past	17.3	9.6	9.6	
	Non	27.8	49.9	57.6	
Drinking status	Yes	57	49.8	56.8	0.001
	No	43	50.2	43.2	
Aerobic exercise	Yes	59.9	57.6	56	0.126
Resistance exercise	Never	71.4	73	71.2	0.007
	Mid	14.7	17.1	18.7	
	High	13.9	9.9	10.1	
Lung function	FVC	3.66 ± 0.04 ^a^	2.85 ± 0.03 ^b^	3.67 ± 0.01 ^a^	<0.001
	FVCp (% predicted)	90.92 ± 0.55 ^a^	73.60 ± 0.26 ^b^	95.73 ± 0.14 ^c^	<0.001
	FEV1	2.31 ± 0.03 ^a^	2.30 ± 0.02 ^a^	2.94 ± 0.01 ^b^	<0.001
	FEV1/FVC	0.63 ± 0.00 ^a^	0.81 ± 0.00 ^b^	0.80 ± 0.00 ^c^	<0.001
SMI		6.94 ± 0.04 ^a^	7.03 ± 0.05 ^a^	6.82 ± 0.02 ^b^	<0.001
Comorbidities					
Hypertension		46.2	49.5	38.6	<0.001
Diabetes		40	48	32.9	<0.001
High triglyceride		37	44.2	34.1	<0.001
Low HDL-C		46.3	53.2	45.7	<0.001
Abdominal obesity		29.7	43.5	29.9	<0.001

Data are presented as means ± SE or numbers (%). BMI, body mass index; FVC, forced vital capacity; FEV1, forced expiratory volume in 1 s. ^a,b,c^ The same letters indicate non-significant differences between groups based on the Bonferroni multiple comparison test.

**Table 2:** Characteristics of participants according to LMS.

Factors	Categories	LMS (*n* = 2623)	Normal (*n* = 6357)	*p*
		M or %	M or %	
Prevalence (COPD/RPD)		15.3/10.2	9.6/11.2	<0.001
Age		55.65 ± 0.30	53.40 ± 0.18	<0.001
Sex	Male	35.1	54.7	<0.001
Education	Elementary	16.6	12.9	<0.001
	Middle	21.8	19.9	
	High	26	24.6	
	University	35.7	42.6	
Marital status	With	82.6	86.8	<0.001
	Without	17.4	13.2	
Personal income	Q1 (Lowest)	24.3	24.2	0.969
	Q2	26.2	26.5	
	Q3	25.3	25.7	
	Q4 (highest)	24.2	23.7	
BMI	Low	5.4	0.1	<0.001
	Normal	87.4	50.7	
	Overweight	6.9	44.6	
	Obesity	0.3	4.6	
Smoking status	Current	28.3	39	<0.001
	Past	7.7	11.5	
	Non	63.9	49.6	
Drinking status	Yes	47.6	59.2	<0.001
	No	52.4	40.8	
Aerobic exercise	Yes	54.1	57.5	0.019
Resistance exercise	Never	76.9	69.3	<0.001
	Mid	16	18.8	
	High	7.1	11.8	
Lung function	FVC	3.29 ± 0.02	3.69 ± 0.01	<0.001
	FVC (% predicted)	92.77 ± 0.31	92.79 ± 0.20	0.968
	FEV1	2.54 ± 0.02	2.89 ± 0.01	<0.001
	FEV1/FVC	0.77 ± 0.00	0.79 ± 0.00	<0.001
SMI		5.73 ± 0.02	7.28 ± 0.02	<0.001
Hypertension		27.6	38.5	<0.001
Diabetes		35.1	42.7	<0.001
High triglyceride		11.4	38.8	<0.001
Low HDL-C		43.3	47.8	<0.001
Abdominal obesity		28.7	37.9	<0.001

Data are presented as means ± SE or numbers (%). BMI, body mass index; FVC, forced vital capacity; FEV1, forced expiratory volume in 1 s.

**Table 3:** Odds ratios for COPD and RPD according to LMS status.

	Lung Function	LMS	
		OR (95% CI)	*p*
Crude	Normal	1 (reference)	
	COPD	1.701 (1.432–2.020)	<0.001
	RPD	0.969 (0.814–1.154)	0.724
Model 1	Normal	1 (reference)	
	COPD	1.791 (1.472–2.178)	<0.001
	RPD	0.985 (0.824–1.177)	0.864
Model 2	Normal	1 (reference)	
	COPD	1.519 (1.225–1.883)	<0.001
	RPD	1.206 (0.982–1.883)	0.074
Model 3	Normal	1 (reference)	
	COPD	1.543 (1.246–1.910)	<0.001
	RPD	1.225 (0.997–1.505)	0.054

Model 1: age, sex, education, marital status, personal income; Model 2: Model 1 + BMI, smoking, drinking, aerobic, resistance exercise; Model 3: Model 2 + comorbid conditions.

**Table 4:** Odds ratios for COPD and RPD according to SMI.

	Lung Function	SMI	
		OR (95% CI)	*p*
Crude	Normal	1 (reference)	
	COPD	1.087 (1.021–1.158)	<0.001
	RPD	1.161 (1.081–1.246)	<0.001
Model 1	Normal	1 (reference)	
	COPD	0.552 (0.494–0.617)	<0.001
	RPD	1.053 (0.947–1.172)	0.338
Model 2	Normal	1 (reference)	
	COPD	0.723 (0.628–0.832)	<0.001
	RPD	0.921 (0.804–1.056)	0.237
Model 3	Normal	1 (reference)	
	COPD	0.713 (0.619–0.821)	<0.001
	RPD	0.909 (0.793–1.042)	0.170

Model 1: age, sex, education, marital status, personal income; Model 2: Model 1 + BMI, smoking, drinking, aerobic, resistance exercise; Model 3: Model 2 + comorbid conditions.

## Data Availability

All data were anonymized and can be downloaded from the website (https://knhanes.kdca.go.kr/knhanes, accessed on 15 November 2024).
